# Inflation Developments in the Euro Area Since the Onset of the Pandemic

**DOI:** 10.1007/s10272-022-1032-y

**Published:** 2022-04-08

**Authors:** Christiane Nickel, Gerrit Koester, Eliza Lis

**Affiliations:** Division Prices & Costs, European Central Bank, Sonnemannstrasse 20, 60314 Frankfurt a. Main, Germany

**Keywords:** E31, P44, J30

## Abstract

There is hope that the Russian war on Ukraine could expedite the energy transition in Europe leading to a new and more environmentally sustainable steady state.

The coronavirus (COVID-19) pandemic shock required lockdowns and containment measures in the euro area, which implied a shutdown of businesses and/or an increase in costs for some sectors. The shock was multidimensional, stemming from both external and domestic sources, hitting both demand and supply and affecting both the aggregate and the sector-specific level. At the same time, the pandemic shock was countered by an unprecedented policy response both at the national and the supranational level. In combination, all of this has led to considerable volatility of inflation in the euro area. As measured by the Harmonised Index of Consumer Prices (HICP), headline inflation in the euro area, which had equalled 1.2% in 2019, fell to 0.3% in 2020 and was even negative in the second half of 2020 before increasing again to 2.6% in 2021. Since mid-2021, headline inflation increased particularly sharply and reached a historical high of 5.9% in February 2022. This article discusses the drivers of inflation developments in the euro area since the onset of the pandemic as well as the recent inflation outlook, which has become very uncertain and will crucially depend on how the war in Ukraine will unfold.

## The pandemic has shaped the pattern of inflation in the euro area

Headline inflation as reflected in the HICP – the index underlying the ECB's definition of price stability (Eurosystem work stream on inflation measurement, 2021) – showed a decreasing trend over 2020 and declined from 1.2% in February 2020 (before the start of the pandemic in the euro area) to 0.3% in December 2020 (see Figure 1). The initial decline in headline inflation was mainly due to a fall in the contribution of energy inflation resulting from collapsing oil prices. Headline inflation fell further in the second half of the year as HICP inflation excluding energy and food (HICPX) also increasingly contributed to the disinflationary tendencies, mainly owing to a decline in services inflation and, to a lesser extent, a decline in non-energy industrial goods (NEIG) inflation. This can be linked to pandemic-related restrictions but also to temporary factors like the reduction of the German VAT by three percentage points in the second half of 2020 (O'Brien et al., 2021).

From January 2021 on, headline inflation showed a strong upward trend bringing headline inflation to 5.9% in February 2022. While energy inflation played a key role in this upward trend of headline inflation since autumn 2021, higher NEIG and services inflation as well as higher food inflation in recent months have also been important.

## Energy inflation is currently accounting for more than half of headline inflation

Oil and gas prices fell sharply at the onset of the pandemic.[1] This large drop reflected mainly the negative impact of the pandemic on energy demand (Koester and Rubene, 2021). As a result, the annual rate of change of HICP energy declined markedly, reaching its trough in May 2020 at -11.9%, levels last observed in 2009, and HICP energy inflation contributed negatively to headline inflation through most of 2020 (see Figure 1). Since mid-2020, energy prices started to rise as global demand recovered and supply constraints, especially on the gas market, rose. Consequently, HICP energy increased from its trough in May 2020 to 28.8% in January 2022 – with base effects linked to the previous collapse of oil prices contributing around ten percentage points to HICP energy inflation. Data for the first months of 2022 suggest that the contribution of gas and electricity prices to HICP energy inflation has increased further (see Figure 2), with prices for electricity and gas being reset at the start of the new year in many countries. Overall energy inflation accounted for more

**Christiane Nickel**, European Central Bank, Frankfurt am Main, Germany.

**Gerrit Koester**, European Central Bank, Frankfurt am Main, Germany.

**Eliza Lis**, European Central Bank, Frankfurt am Main, Germany.

1 The immediate decline in the oil price was particularly pronounced as for instance the Brent crude oil price dropped by 75% while the Dutch Title Transfer Facility (the Dutch trading hub for gas and the main reference hub for gas trading in Europe) gas price fell by 44% between February and April 2020.

Figure 1
**Headline inflation and its main components**
annual percentage changes; percentage point contributions

Note: The latest observations are for February 2022.

Sources: Eurostat and ECB calculations.

Figure 2
**Decomposition of HICP energy inflation**
annual percentage changes; percentage point contributions

Note: The latest observations are for February 2022.

Sources: Eurostat and ECB calculations.

than half of headline inflation in February 2022. The Russian war in Ukraine has implied a further soaring in energy prices and energy inflation to a new historical high of 32% in February 2022 and increased the uncertainty about the future path of energy prices and inflation more generally.

Indirectly, higher energy commodity prices also feed in through the pricing chain via higher input costs to food, non-energy industrial goods and services (Koester et al., 2021a). NEIG and services with high energy intensity such as pharmaceutical products or travel services can be particularly affected. The rising energy costs have likely contributed to increases in food inflation and non-energy industrial goods inflation – which stood at 4.1% and 3.0% respectively in February. Yet, this pass-through takes time and unfolds over years, implying that rising energy prices will likely push up food and underlying inflation in the future.

### External factors have played a key role for inflation developments in the euro area

Already now, the largest part of inflation in the euro area reflects shocks generated abroad, via net imports of energy and commodities or via the import content of other goods and services. This can be illustrated by decomposing HICP inflation into energy and food as well as into items with a high and a low import content (Figure 3). Inflation in items with low import content, for which domestic price pressures play a key role, has been much lower than for HICP and also HICPX. This contrasts with the period prior to the pandemic where the shocks driving inflation were more equally distributed and reflected both external as well as domestic factors.

## Underlying inflation increasing and broadening

Indicators of underlying inflation, which signalled low inflation in the euro area for an extended period from 2013 to 2019 (Koester et al., 2021b) have remained subdued since the start of the pandemic before picking up strongly over recent months.

As no single measure of underlying inflation has proved superior, the ECB is usually monitoring a broad range of different indicators (Nickel and O'Brien, 2018; Ehrmann et al., 2018). HICP excluding food and energy inflation rose to 2.7% in February, up from 2.3% in January (Figure 4). Measures of underlying inflation that seek to remove the impact of temporary factors like HICPXX inflation (which, in addition to energy and food, also excludes travel-related items, clothing and footwear), the model-based Persistent and Common Component of Inflation (PCCI) and the Supercore indicator (which comprises cyclically sensitive HICP items), have tended to edge upwards in recent months. While all indicators of underlying inflation have moved above 2%, this probably reflects – at least in part – the indirect effects of elevated energy prices.

Price pressures have been broadening across the spectrum of goods in the euro area (see Figure 5). This is reflected by the fact that the share of items with inflation

Figure 3

**Contributions from items with high and low import content to HICP inflation**

annual percentage change and percentage point contributions

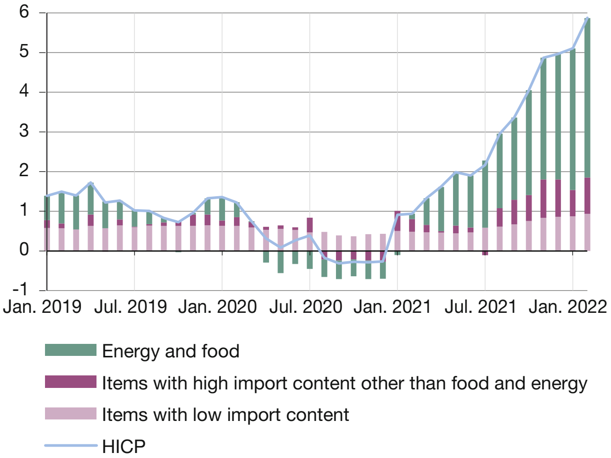

Notes: Items with low import content refer to the items in the HICP that are characterised by a direct and indirect import content in consumption expenditure of less than 15%. Decomposition based on Fröhling et al. (2021). Latest observations: February 2022.

Sources: Eurostat, WIOD and ECB staff calculations.

rates above 4% (accounting for 24% of items in December) and of items with inflation rates between 2% and 4% (accounting for 44%) has strongly increased. A year ago – in February 2021 – these shares were much lower and equalled 6% (share of items with inflation rates above 4%) and 10% (share of items with inflation rates between 2% and 4%).

## Supply bottlenecks and reopening effects have played a key role

The pattern observed in underlying inflation in the euro area can be linked to developments in the real economy with a large decline in global economic activity in 2020 followed by a significant recovery starting in the third quarter of 2020 and a boost from the rollout of vaccine programmes since late 2020 (Lane, 2021a). This is in line with the finding that while the assessment of the euro area Phillips curve (linking inflation to developments in economic slack) has become more complicated due to numerous confounding factors during the pandemic, it is still at play – even if it is hard to pin down precisely (Bobeica et al., 2021).

The economic recovery has remained asymmetric as social distancing has constrained demand and activity levels in high-contact service sectors, especially travel re-

Figure 4

**Indicators of underlying inflation**

annual percentage changes

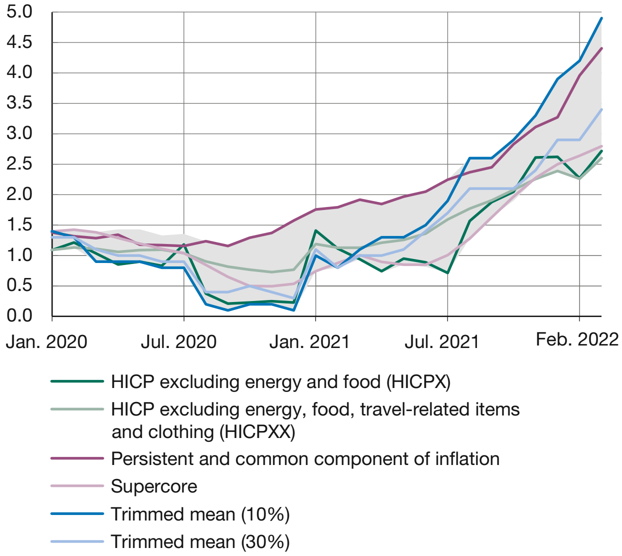

Notes: The range of indicators of underlying inflation includes HICP excluding energy, HICP excluding energy and unprocessed food, HICPX, the 10% and 30% trimmed means, and the weighted median. The latest observations are for February 2022.

Sources: Eurostat and ECB calculations.

lated services, for quite some time with a direct impact on service inflation (Lis and Nordeman, 2021). At the same time, a relative expenditure switch towards the consumption of goods has strongly increased demand for goods. In addition, manufacturing production continued to be

Figure 5

**Distribution of inflation rates across items included in HICPX inflation**

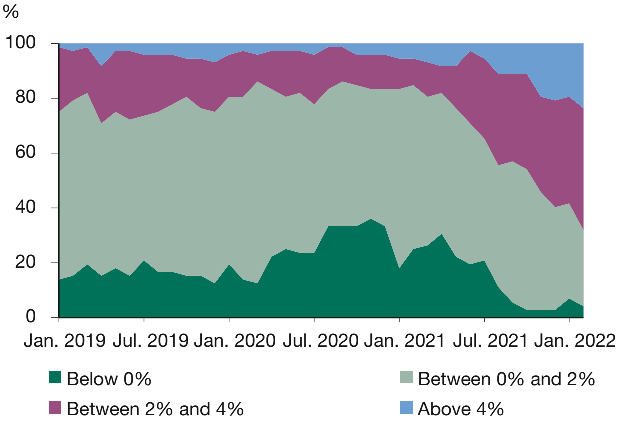

Note: Latest observation February 2022.

Sources: Eurostat and ECB calculations.

Figure 6

**Decomposition of NEIG inflation – the impact of pandemic-related effects**

annual percentage change and percentage point contributions

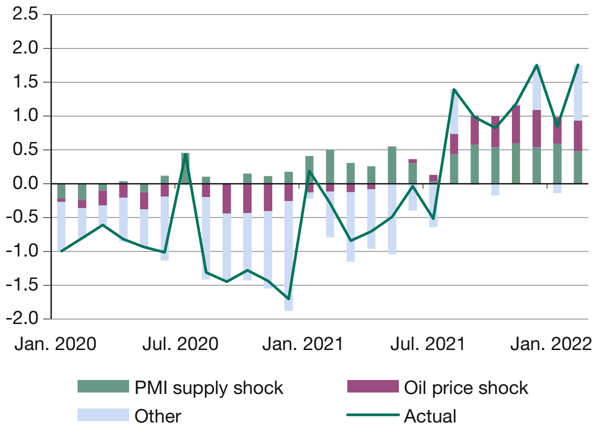

Note: All series are demeaned. Latest observations: January 2022.

Sources: Eurostat, NIPE and ECB staff calculations.

Figure 7

**High-contact and low-contact services inflation**

annual percentage change, at constant 2020 weights; percentage point contributions

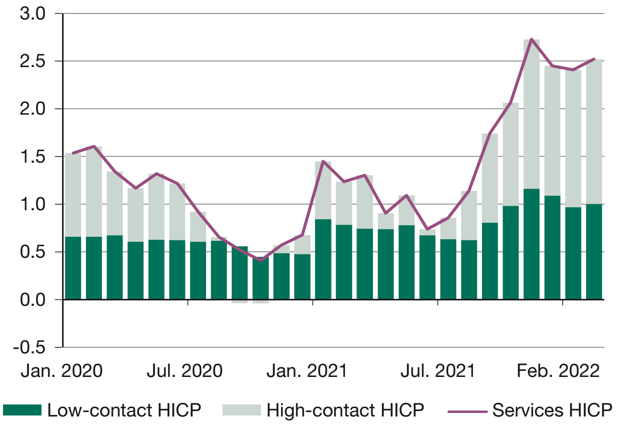

Note: Series are shown with constant 2020 weights. Latest observation: December 2021.

Sources: Eurostat and ECB staff calculation.

disrupted by the shocks to labour supply and temporary factory shutdowns as well as lack of transportation capacities around the world, leading to an important role of bottlenecks for inflation in goods.[2]

Zooming in on developments in NEIG inflation, the rates of around 2.5%-3.0% seen since mid-2021 are well above long-term averages of NEIG inflation (0.6%) – with supply bottlenecks and high energy costs playing an important role. Estimates based on a vector autoregression (VAR) model including a supply shock derived based on PMI supply delivery times as an indicator for supply bottlenecks and oil prices indicates that these two factors contributed around one percentage point to NEIG inflation over the last few months (see Figure 6).[3] Other factors contributing to the relatively high NEIG inflation include the shift from services to goods demand.

Turning to services, the strong recovery in inflation has been driven especially by high-contact services, while low-contact services increased only very moderately (Figure 7). This reflects a key role of re-opening effects linked to the easing of the containment measures as well as an impact of the energy price increase – which pushes up costs for transportation, which are also part of the high-contact services.

## Wage growth has remained muted and longer-term inflation expectations have re-anchored

Wage growth – a major driver of services inflation in the euro area – has remained moderate thus far. Important measures of wage growth like compensation per employee or compensation per hour have been heavily affected by the changing impact of government support measures related to job retention schemes (Dias da Silva et al., 2020). Workers maintained their employment status but actual hours worked per person declined and workers only received part of their usual compensation, lowering compensation per employee while annual growth in compensation per hour increased. Over the course of the pandemic, the divergence between compensation per employee growth and compensation per hour growth was strongly affected by the importance of job retention schemes. The distortion from these schemes has declined recently and can be expected to remain moderate looking ahead – but will not entirely disappear until the pandemic is fully over and these support schemes are not needed anymore. This causes some difficulty in interpreting developments in wage measures like compensation per hour or per employee.

2 A key role of supply bottlenecks and reopening effects for underlying inflation has also been observed in other jurisdictions – see e.g. Cuquerella Ricarte et al. (2022) and Koester et al. (2021c).

3 Historical decompositions are based on a VAR including a bottleneck proxy, oil prices, HICP NEIG, producer prices, industrial production, export and import volumes. The bottleneck and oil price shock are identified using short-run restrictions averaged across all possible orderings of the bottleneck and oil price series. As a bottleneck proxy, a PMI supply shock is estimated from a separate VAR including PMI output and supply deliver times and identified via sign restrictions. Gas prices are not explicitly included in the model, but oil prices are highly correlated with gas prices, and thus capture a major part of the energy shock.

Figure 8

**Breakdown of compensation per employee into compensation per hour and hours worked**

annual percentage changes

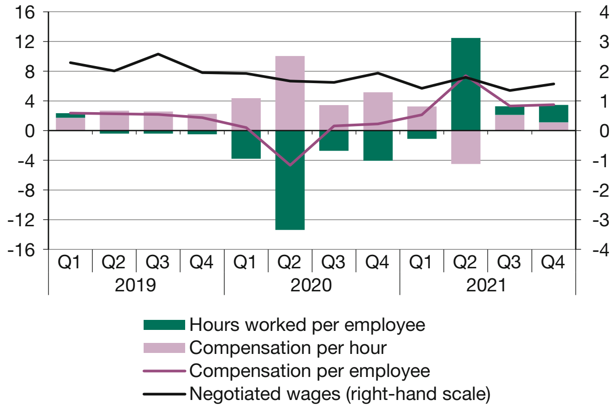

Note: The latest observations are for the fourth quarter of 2021.

Sources: Eurostat and ECB calculations.

Figure 9

**Survey-based indicators of inflation expectations**

annual percentage changes

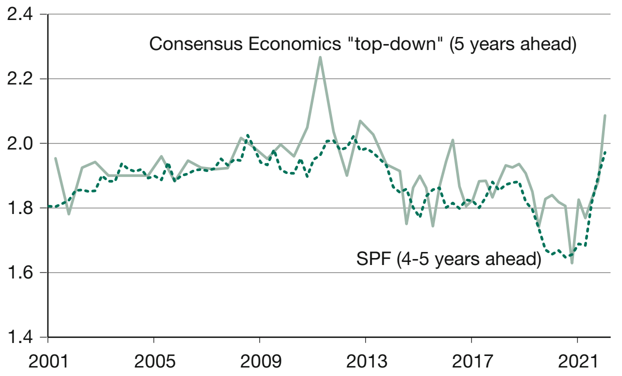

Note: Latest observations: The Survey of Professional Forecasters for the first quarter of 2022 was conducted between 7 and 13 January 2022; long-term forecasts from Consensus Economics were released on 13 January 2022.

Sources: Consensus Economics and ECB Survey of Professional Forecasters.

Therefore, signals from indicators of negotiated wages, which have not been directly affected by developments in hours worked or the recording of benefits from job retention schemes introduced in response to the pandemic, are especially important at the current juncture (Koester et al, 2020). Growth in negotiated wages remained moderate in the fourth quarter of 2021, standing at 1.5%, up from 1.4% in the previous quarter and averaging 1.5% over 2021 as a whole, down from 1.8% in 2020. Looking ahead, one key question is to what extent the current surge in inflation will also push up wage demands and wage agreements in the euro area (in the form of so-called second-round effects). While the prevalence of formal and informal wage indexation to inflation has been reduced since the global financial crisis and is only relatively limited in the euro area (Koester and Grapow, 2021), high inflation is still likely to be an important aspect for wage negotiations. In this context, a recent ECB survey of large European companies indicates that wage growth in 2022 could be somewhat stronger, with some respondents citing the current high levels of inflation as a contributing factor (Gareis et al., 2022; Lane, 2022).

Over the medium to long term, inflation expectations play a key role in the achievement of a central bank's inflation target. Inflation expectations that are firmly anchored in line with the inflation target support the achievement of that goal by guiding wage- and price-setting decisions in the economy. Deviations of inflation expectations from the inflation target may become self-reinforcing (Eurosystem work stream on inflation expectations, 2021).

The pandemic led to a fall in longer-term inflation expectations in the euro area to historically low levels in 2020. During 2020, long-term survey-based inflation expectations based on the ECB Survey of Professional Forecasters (SPF) stayed at a narrow range of 1.64%-1.67%. Since the beginning of 2021, longer-term inflation expectations have been moving up, indicating that long-term inflation expectations across a range of measures have re-anchored at the ECB's inflation target. This is reflected, for example, in the ECB's Survey of Professional Forecasters (SPF) for the first quarter of 2022 (which was conducted in the second week of January) and the January 2022 Consensus Economics forecasts, in which longer-term inflation expectations have risen to 2.0% and 2.1% respectively, up from 1.9% in their respective previous survey rounds (Figure 9). This should also contribute further to underlying inflation and help headline inflation to settle durably at the 2% target of the ECB. Looking ahead, it will be key to closely monitor the potential effects of the current spike in inflation on the evolution of indicators of longer-term inflation expectations in particular. This is even more important given the finding that households' inflation expectations tend to be influenced strongly by the prices of goods that they purchase frequently (D'Acunto et al., 2022) such as fuel and groceries – which currently record very high inflation rates.

## The outlook for inflation in the euro area

Over recent months, headline inflation has soared significantly, reflecting mainly a sequence of energy price

Figure 10
**Euro area HICP outlook – baseline, adverse and severe scenario**
annual percentage change

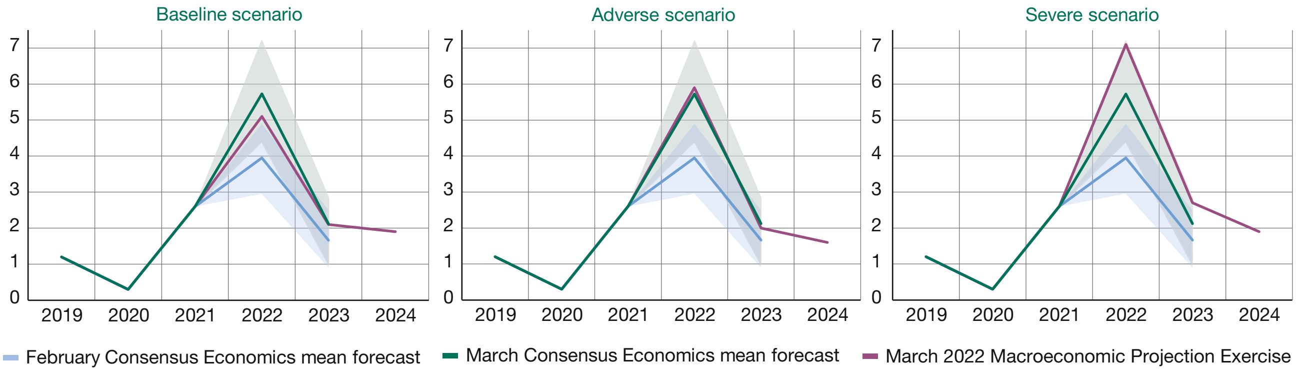

Note: The panels show the baseline, adverse and severe scenarios of the March 2022 ECB staff macroeconomic projections for the euro area.

Sources: Eurostat, Consensus Economics and ECB staff calculations.

shocks, but also global supply bottlenecks and re-opening effects have contributed to inflationary pressures. These turned out to be more persistent than previously expected by most forecasters. Still, these factors are assessed to be the result of transitory disturbances and pandemic-related adjustments and, hence, are expected to fade over the coming years.

While the Russian war in Ukraine has intensified the uncertainty on energy prices, oil and gas price future curves suggest a decline in energy prices, which implies sharp declines in energy inflation looking ahead. Similarly, global supply disruptions might increase in the near term, again due to shortages of key inputs from Russia, and only fade later easing the pressure on non-energy industrial goods inflation. As more and more containment measures are lifted across Europe, price pressures in high-contact services are likely to ease. The Russian war in Ukraine might have some dampening effects on inflation via the negative growth effect on underlying inflation in the euro area, but these are likely to be offset by indirect effects from the higher energy prices triggered by the conflict.

So, while inflation in the euro area is expected to stay high this year as inflationary pressures have broadened and become more persistent, inflation is likely to stabilise at 2% in the medium term. This is also reflected in the latest ECB (2022) staff projections – in which the baseline foresees inflation equal to 5.1% in 2022 before falling to 2.1% in 2023 and 1.9% in 2024. An adverse scenario and a severe scenario, however, expect substantially higher inflation in 2022 and 2023 but a moderation of inflation to 1.9% (in the severe scenario) or even 1.6% (in the adverse scenario) in 2024 (Figure 10).

Looking further ahead, structural changes are likely to play an important role for inflation in the euro area.[4] Climate change and climate policies in particular are likely to impact energy prices, changes in relative prices and the dynamics in overall inflation. The overall impact is subject to high uncertainties in terms of sign, size and timing. The Russian war in Ukraine has increased this uncertainty even further. Until around the end of this decade, upward pressures on consumer energy prices and increased energy price volatility are possible, mainly due to higher taxes and enduring high dependence on fossil fuels. Around the end of this decade, upward pressure might ease, especially for electricity prices as the share of renewables in electricity production is expected to grow fastest. For transport and heating, fossil energy sources will still dominate beyond this decade and changes in energy commodity prices will continue to have a significant impact on HICP energy inflation. Yet, their relative importance should gradually decline with increasing electrification based on renewable sources and increasing energy efficiency. There is hope that the Russian war on Ukraine could expedite the energy transition in Europe leading to a new and more environmentally sustainable steady state.

4 See also the discussion in part 5 of Koester et al. (2021b).
